# Guided Bone Regeneration in the Treatment of Lateral Periodontal Cysts

**DOI:** 10.7759/cureus.83937

**Published:** 2025-05-12

**Authors:** Vithleem Xanthopoulou, Dimitrios Xanthopoulos, Sofia Zarenti, Konstantinos Papadimitriou, Leonidas Batas, Eleftherios Anagnostou, Lazaros Tsalikis

**Affiliations:** 1 Department of Preventive Dentistry, Periodontology and Implant Biology, Aristotle University of Thessaloniki, Thessaloniki, GRC; 2 School of Dentistry, National and Kapodistrian University of Athens, Athens, GRC; 3 Department of Oral Pathology and Stomatology, Aristotle University of Thessaloniki, Thessaloniki, GRC

**Keywords:** biopsy, bone regeneration, odontogenic cysts, oral pathology, periodontal cyst

## Abstract

The lateral periodontal cyst (LPC) is a rare, noninflammatory developmental odontogenic cyst typically associated with the roots of vital teeth. Often asymptomatic, LPCs are frequently discovered incidentally during routine radiographic examinations. This case report presents the diagnosis and treatment of an LPC in a 56-year-old female and discusses relevant diagnostic and therapeutic considerations. A 56-year-old female patient, presenting for a routine recall appointment, was found to have a radiolucent area lateral to the mesial root of tooth #44 on radiographic examination. Clinical findings were unremarkable, suggesting a presumptive diagnosis of an LPC. Due to a subsequent crown failure rendering the tooth unrestorable, surgical intervention involved simultaneous extraction of tooth #44, complete enucleation of the cystic lesion, and guided bone regeneration (GBR) to address the resultant bone defect. A biopsy was obtained for definitive histopathologic confirmation. Histopathologic analysis confirmed the diagnosis of a benign cyst consistent with the characteristics of an LPC. The patient was monitored for six months postoperatively, with both clinical and radiographic evaluations showing no evidence of recurrence, indicating a successful outcome with complete bone fill in the treated area. In summary, LPC is an uncommon and often asymptomatic lesion requiring histological confirmation for accurate diagnosis. Surgical removal, coupled with bone regeneration techniques like GBR to manage post-enucleation defects, offers a favorable prognosis with a low risk of recurrence. Early detection through routine radiographic screening remains crucial for timely intervention and optimal patient outcomes.

## Introduction

The lateral periodontal cyst (LPC) is an infrequent, non-keratinized, and non-inflammatory developmental odontogenic cyst that predominantly occurs lateral to the roots of vital teeth. It can also arise along the lateral periodontium or between the roots of erupted vital teeth within the alveolar bone [1,2]. Representing approximately 0.4% of all odontogenic cysts [1,3], LPCs are typically asymptomatic, often lacking pain or other clinical signs, and are usually identified during routine radiographic examinations. However, gingival swelling may be observed in some instances. The mandible’s canine and premolar regions are the most common sites for LPC development, followed by the anterior maxilla [4,5]. While LPCs are most frequently reported in the fourth to seventh decades of life, no specific gender or racial predilection has been established [5]. Radiographically, an LPC typically appears as a well-defined radiolucent area adjacent to a vital tooth’s root, with its size ranging from a few millimeters to encompassing the entire lateral aspect of the root [4,6]. The histogenesis of LPC epithelial lining remains a subject of debate [7], with potential origins including rests of the dental lamina, rests of Malassez, or reduced enamel epithelium [1,5,7,8]. Histologically, LPCs are characterized by a thin, non-keratinized epithelium supported by connective tissue resembling the reduced enamel epithelium [9]. Inflammatory cells are typically absent, although glycogen-rich cells and focal epithelial thickenings are often present. LPCs are classified as either unicystic or multicystic, the latter being termed a botryoid odontogenic cyst [10]. The standard treatment involves surgical enucleation and subsequent monitoring for recurrence, which is rare, with follow-ups ranging from six months to one year [3,11]. In situations where simple enucleation could potentially compromise the periodontal health of the adjacent tooth due to the remaining defect, combining enucleation with guided bone regeneration (GBR) has been shown to minimize the risk of recurrence and promote bone fill [12,13]. This paper aims to present a case of LPC treated with surgical enucleation and primary bone defect repair utilizing GBR with a xenograft and a resorbable collagen membrane. This case report adheres to the 2013 CARE Guidelines.

## Case presentation

Patient information

**Figure 1:**
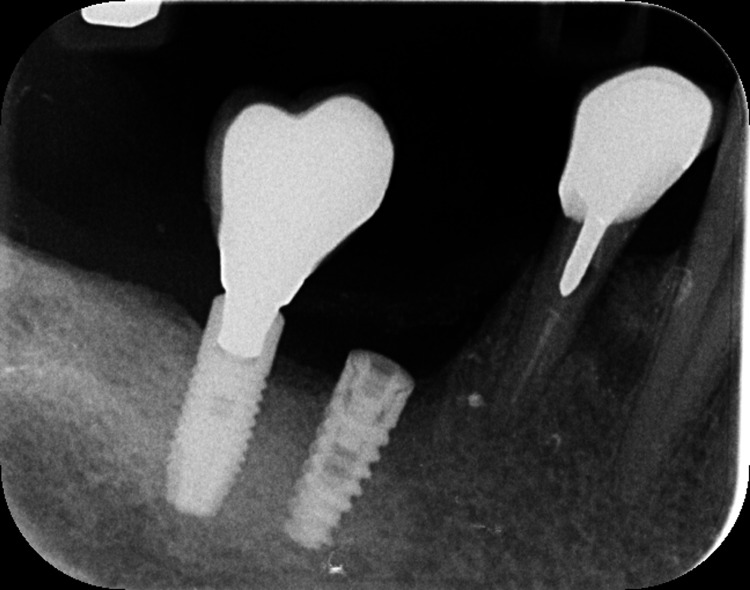
Preoperative radiograph showing LPC adjacent to tooth #44 LPC, lateral periodontal cyst

A 56-year-old female patient with no significant medical history presented to the Department of Preventive Dentistry, Periodontology, and Implant Biology at the Dental School, Aristotle University of Thessaloniki in Thessaloniki, Greece, for a six-month recall appointment. Routine radiographic examination revealed a well-defined radiolucent area located between the mandibular canines and first premolars (Figure 1). The lesion was asymptomatic, and periodontal probing of the adjacent tooth revealed a depth of 3.0 mm with no mobility. The initial diagnosis was an LPC. One week later, the patient reported a failure of the crown on tooth #44. Clinical examination confirmed the tooth was unrestorable, leading to the decision for extraction.

Surgical procedure 

**Figure 2:**
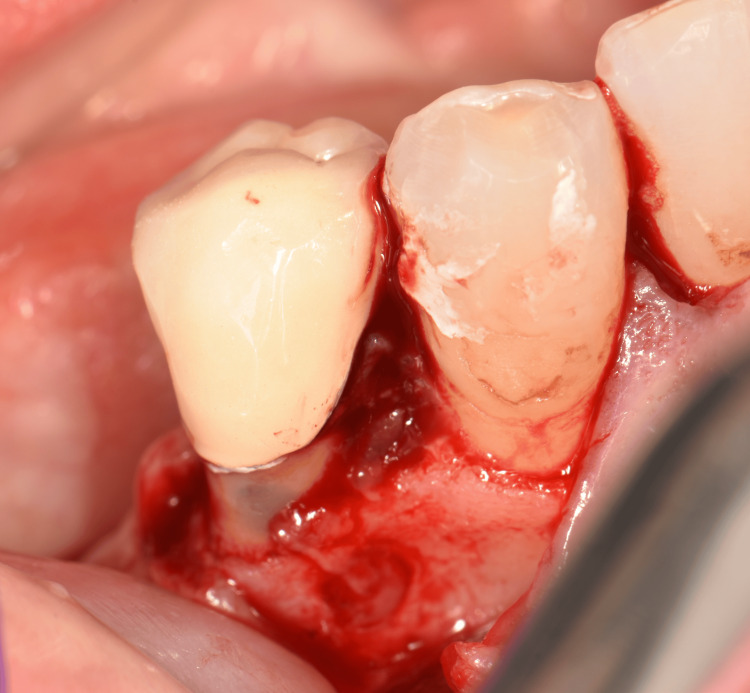
Intrasulcular incision with anterior vertical releasing incision

**Figure 3:**
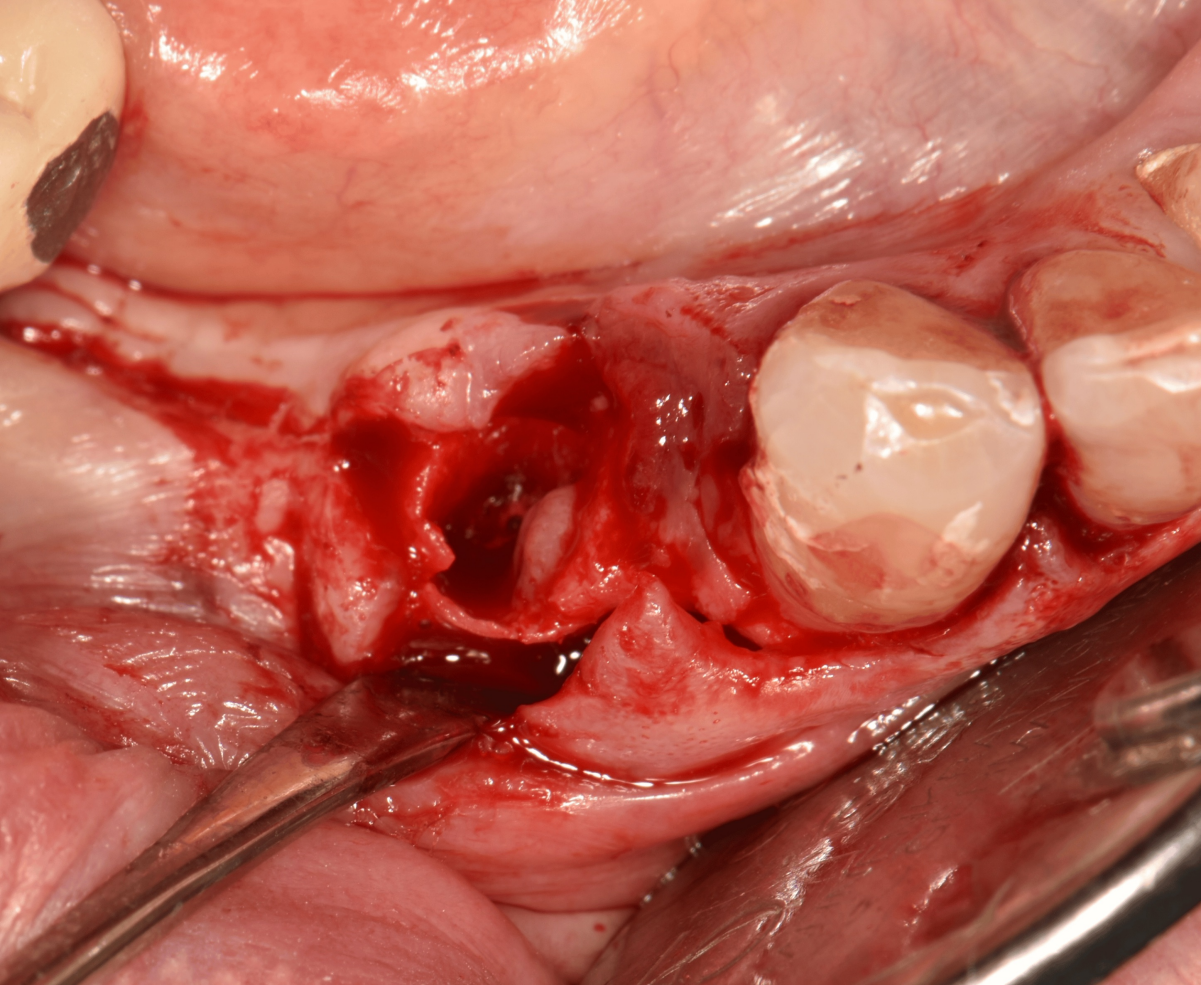
Tooth extraction and surgical enucleation of the lesion located 3.0 mm from the bone crest

**Figure 4:**
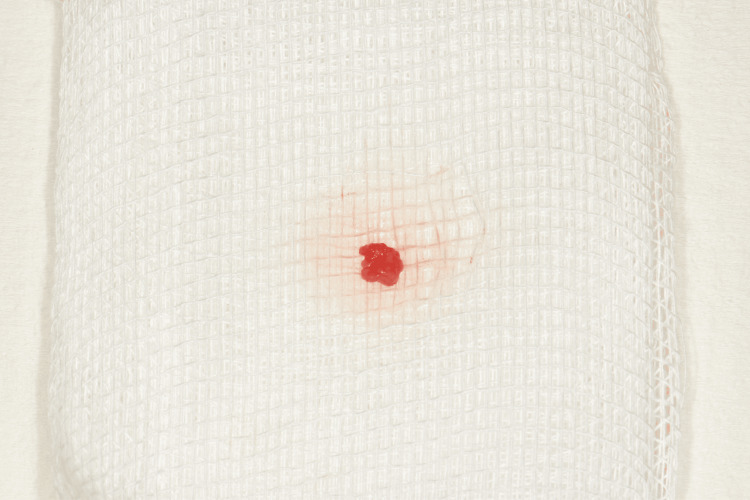
LPC LPC, lateral periodontal cyst

**Figure 5:**
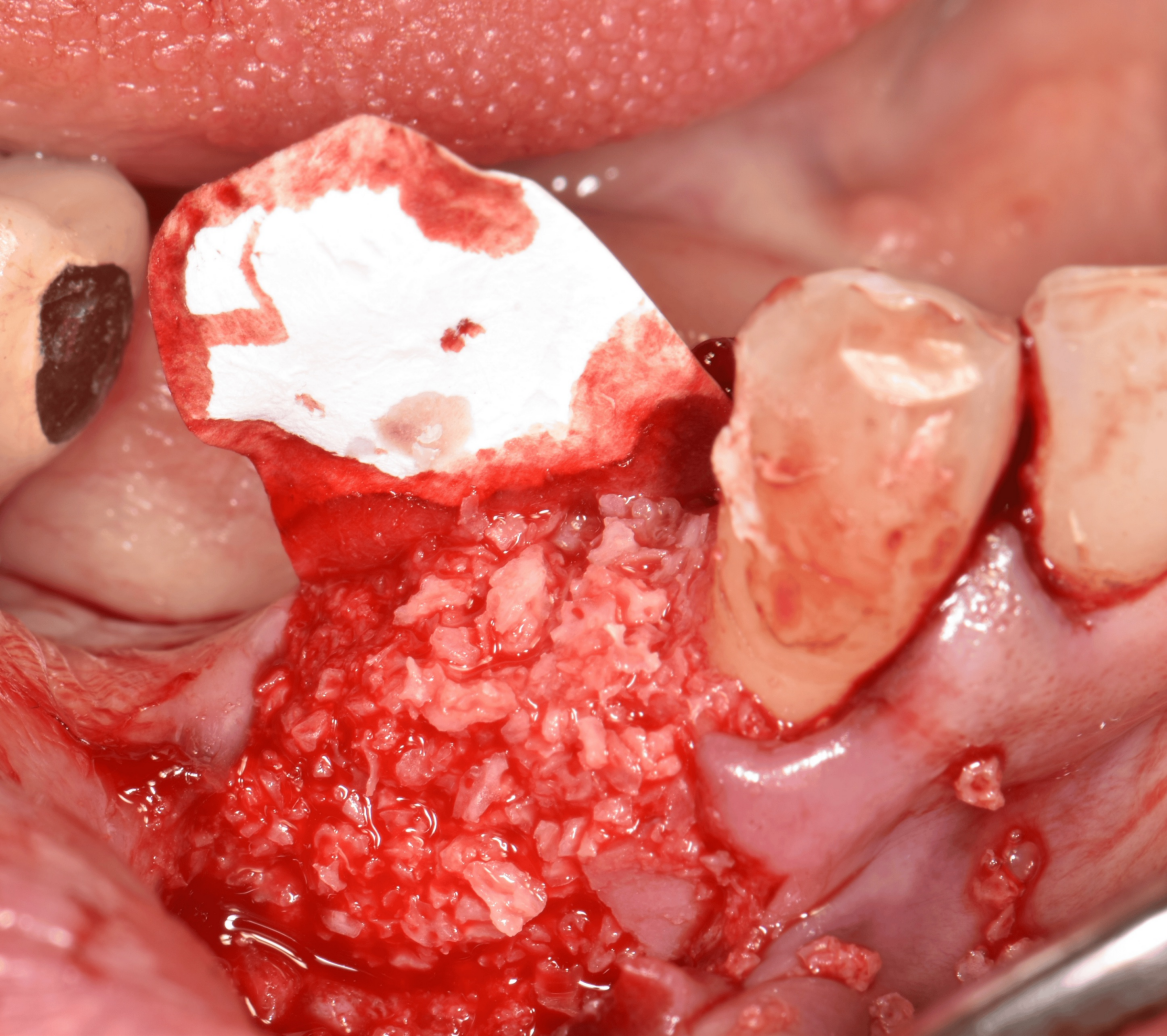
Bone regeneration with allograft (FDBA – corticocancellous granules) covered by a resorbable collagen membrane

**Figure 6:**
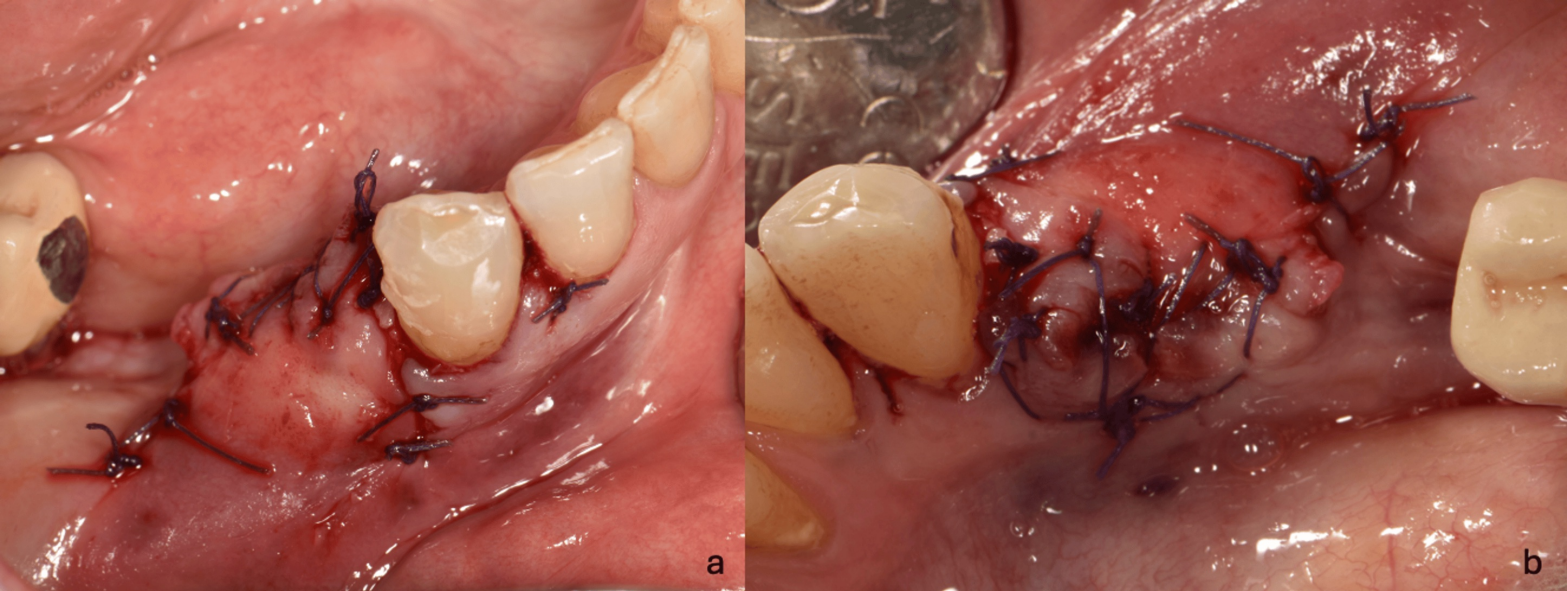
Single interrupted sutures for re-approximation of the flap: (a) buccal and (b) lingual view

Based on the clinical and radiographic diagnosis of an LPC and following informed consent, a treatment plan involving simultaneous extraction of tooth #44, surgical enucleation of the lesion, and GBR was implemented. Under local anesthesia, an intrasulcular incision with a vestibular mucoperiosteal flap elevation and an anterior vertical releasing incision was performed to optimize lesion visualization. The lesion was noted to be located 3.0 mm apical to the bone crest (Figure 2). Following tooth extraction, the lesion was meticulously enucleated using a Lucas and surgical curette (Figure 3, Figure 4). The resulting defect, encompassing both the extraction socket and the cyst cavity, was thoroughly curetted and irrigated with sterile saline. Subsequently, the defect was filled with an allograft (FDBA - corticocancellous granules, Princeton, NJ, USA) and covered with a resorbable collagen membrane (Remaix Absorbable Membrane 15 mm Å~ 20 mm, Wolhusen, Switzerland) (Figure 5). The mucoperiosteal flap was then repositioned and secured with single interrupted sutures using 4-0 absorbable polyglycolic acid suture (Greece) (Figure 6a, Figure 6b). Postoperatively, the patient was prescribed amoxicillin/clavulanic acid 1,000 mg twice daily for five days (Augmentin-BID 10 tablets, GlaxoSmithKline, London, England) as a prophylactic measure against infection. The postoperative healing period was uneventful, with complete and uncomplicated surgical wound closure.

Histopathological examination

**Figure 7:**
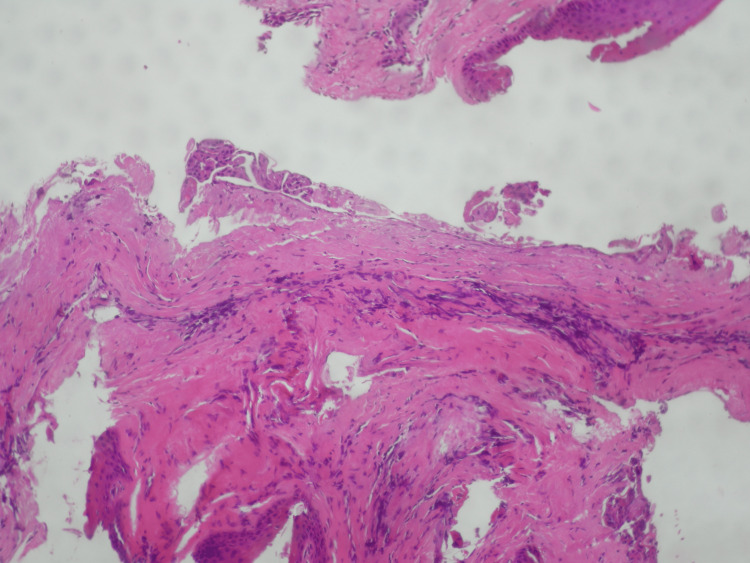
The cystic wall was composed of fibrous tissue, and it was lined by stratified squamous, non-keratinized epithelium (H&E ×100)

**Figure 8:**
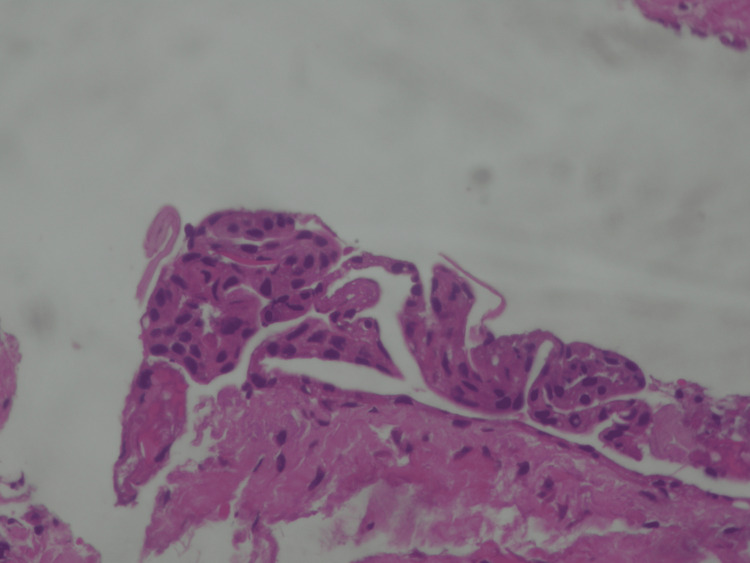
The lining epithelium of the cyst does not show atypia or mitoses (H&E ×400)

The excised specimen was fixed in 10% formalin and submitted to the Oral Pathology Laboratory at the Aristotle University of Thessaloniki for histopathologic analysis. Macroscopic examination revealed the lesion to be 0.5 cm in diameter. Microscopic evaluation of tissue sections demonstrated a benign cystic lesion consistent with the histopathological features of an LPC. The cystic lining consisted of stratified, non-keratinized squamous epithelium (H&E stain, original magnification ×100) (Figure 7). The epithelial cells exhibited oval to round nuclei, with no evidence of atypia or mitotic activity (Figure 8). Additionally, higher magnification (×400) revealed a mild lymphocytic infiltrate within the cystic wall. Based on these findings, the final histopathologic diagnosis was LPC.

Follow-up

**Figure 9:**
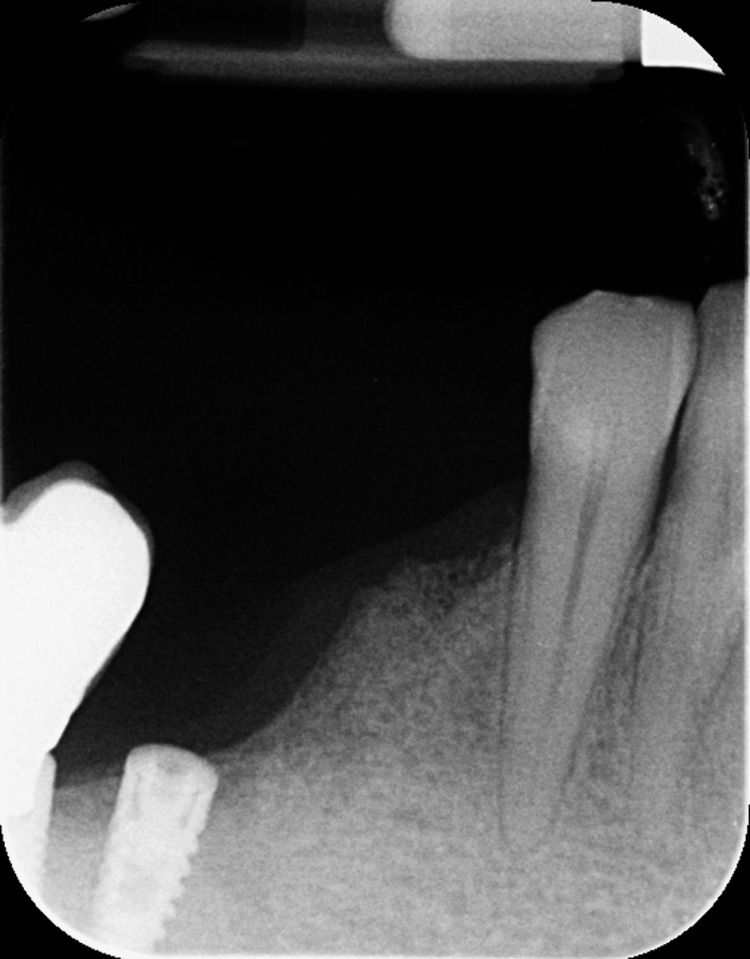
Six months after the healing

Six months after the cyst enucleation and the GBR, the absence of clinical symptoms and a periapical radiograph confirmed satisfactory bone regeneration and absence of recurrence (Figure 9).

## Discussion

The LPC is an uncommon, noninflammatory lesion associated with the root of a vital tooth, typically presenting without clinical signs or symptoms. Infrequently, a minor gingival swelling may be observed. Diagnosis is primarily based on routine radiographic examination, as seen in the presented case [1,2,14]. Accurate differential diagnosis is crucial to avoid misdiagnosis and inappropriate treatment [14]. Key differential diagnoses include odontogenic keratocysts, known for their potential for recurrence and aggressive behavior, requiring differentiation from the typically benign LPC; gingival cysts (epulis cysts), which occur in the soft tissues of the gingiva; lateral radicular cysts, associated with non-vital teeth and sometimes difficult to distinguish from LPCs; pseudocysts, which lack an epithelial lining; and various odontogenic tumors that can present as radiolucent lesions [15]. This report presents a case of LPC, detailing its clinical, radiographic, and histopathologic features, along with the chosen treatment approach. Radiographic examination revealed a well-defined radiolucent area smaller than 1 cm, located lateral to the mesial root of tooth 44. The pathogenesis of LPC is thought to involve the reduced enamel epithelium, given the presence of non-keratinized epithelium. However, the presence of glycogen-rich cells suggests a possible origin from remnants of the dental lamina, and its proximity to the root surface could indicate origin from cell rests of Malassez [1,15]. The standard treatment for LPC is surgical enucleation and curettage, allowing for spontaneous bone healing [3]. In this case, due to the extraction of tooth 44, the treatment involved enucleation of the cyst and management of the postextraction socket and residual bone defect using GBR principles. The bone defect was filled with allograft and covered with a resorbable collagen membrane. Allografts are osteoconductive, providing a scaffold for bone regeneration. Collagen membranes act as barriers, preventing unwanted cell migration and facilitating controlled bone or soft tissue regeneration. Nart et al. [16] demonstrated radiographic bone fill following GTR and bone grafting for LPC treatment at 7 months postoperatively. Subramaniam et al. [17] treated an intraosseous cystic cavity with PRP without grafting. While PRP’s effectiveness in periodontal regeneration can vary [18,19], GTR principles, as employed with allograft and a membrane in this case, can lead to successful outcomes in LPC treatment. In cases involving LPCs, GBR supports bone fill and prevents collapse of surrounding soft tissue by using barrier membranes to exclude epithelial cells and facilitate selective cell repopulation. The combination of osteoconductive graft materials and resorbable membranes promotes predictable bone regeneration. This approach has been successfully demonstrated in similar cases, such as the one reported by Ramalingam et al., where GBR led to complete radiographic bone fill post-LPC treatment [20].

## Conclusions

The LPC is a rare developmental odontogenic cyst predominantly found in the mandible between the roots of premolars and canines, typically affecting individuals in their fourth to seventh decades and often presenting asymptomatically. Accurate diagnosis relies on thorough clinical and radiographic evaluation, with histopathological examination being essential for confirmation. Surgical enucleation, with or without GBR, represents the most effective treatment approach. However, further long-term clinical trials with larger cohorts are needed to definitively assess the efficacy of various treatment techniques.
